# Early structural brain abnormalities in borderline personality disorder

**DOI:** 10.1017/S0033291725101645

**Published:** 2025-10-13

**Authors:** Pilar Salgado-Pineda, Marc Ferrer, Natalia Calvo, Xavier Costa, Josep-Antoni Ramos-Quiroga, Brenda Tarragona, Juan Duque-Yemail, Cristina Vaciana-Verdaguer, Àlex Rué, Paola Fuentes-Claramonte, Silvia Ferrer, Raymond Salvador, Peter McKenna, Edith Pomarol-Clotet

**Affiliations:** 1 FIDMAG Germanes Hospitalàries Research Foundation, Barcelona, Spain; 2 Centro de Investigación Biomédica En Red de Salud Mental, Instituto de Salud Carlos III (CIBERSAM, ISCIII), Barcelona, Spain; 3 Psychiatry and Legal Medicine Department, Universitat Autònoma de Barcelona (UAB), Barcelona, Spain; 4 Psychiatry Department, Hospital Universitari Vall d’Hebron, Centro de Investigación Biomédica En Red de Salud Mental (CIBERSAM), Barcelona, Spain; 5 Psychiatry, Mental Health and Addictions Group, Vall d’Hebron Institut de Recerca (VHIR), Barcelona, Spain; 6 Fundació Orienta, Grup TLP-Barcelona, Barcelona, Spain; 7 Fundació Hospitalàries Barcelona, Barcelona, Spain; 8 Facultat de Medicina i Ciències de la Salut. Universitat Internacional de Catalunya, Barcelona, Spain

**Keywords:** adolescent, borderline personality disorder, voxel-based morphometry

## Abstract

**Background:**

Structural imaging studies of borderline personality disorder (BPD) have identified regions of reduced and increased cortical volume, as well as volume reductions in the hippocampus and amygdala, although with considerable variability across studies. Examining adolescent patients with the disorder can reduce potential confounding effects such as later development of affective and other comorbid disorders.

**Methods:**

Fifty-one adolescents (48 females, 3 males) with BPD and without comorbid disorders and with 43 matched healthy controls underwent whole-brain voxel-based morphometry (VBM). Hippocampus and amygdala volumes were also measured using conventional volumetric techniques.

**Results:**

At a threshold of *p* = 0.05 corrected, the BPD patients exhibited a cluster of grey matter volume reduction in the left temporo-parietal junction (TPJ). No evidence of volume reductions in the hippocampus or amygdala was found. Comparison between the female-only subsamples (48 BPD patients and 37 controls) yielded similar findings. The cluster of volume reduction in the left TPJ continued to be seen in 37 drug-naïve patients.

**Conclusions:**

According to this study, the initial stage of BPD is characterized by decreased grey matter volume in the left TPJ, a region that is implicated in various aspects of social cognition. Given that the volume loss was detected prior to adulthood, in individuals without comorbidities, and among patients who were drug naïve, this finding could be significant for understanding the developmental trajectory of the disease.

## Introduction

Borderline personality disorder (BPD) affects approximately 1.7% of the adult population, with a marked female preponderance (Gunderson, Herpertz, Skodol, Torgersen, & Zanarini, 2018). It is characterized by intense and volatile emotionality, impulsive, often self-destructive behaviour, and an unstable sense of self (Bohus et al., 2021; Gunderson et al., 2018). Although traditionally considered an adult disorder, there is evidence that BPD can be reliably diagnosed during adolescence (Bohus et al., 2021; Brede, Dippold, Bender, Kröger, & Krischer, 2025; Sharp, 2020; Winsper et al., 2016), something that is reflected in the forthcoming ICD-11, which allows the diagnosis of personality disorders from the age of 12 (Pan & Wang, 2024).

Biological factors are widely suspected in BPD and have been studied from a number of perspectives (Leichsenring et al., 2023; Perez-Rodriguez, Bulbena-cabré, Nia, Zipursky, & Goodman, 2018). One of these is brain structural imaging: early studies of this type documented volume reductions in the hippocampus and amygdala (for meta-analyses see Nunes et al., 2009; Ruocco, Amirthavasagam, & Zakzanis, 2012), and also in cortical regions including the anterior cingulate cortex (ACC), the orbitofrontal cortex (OFC), and the right parietal cortex (Denny, Kober, Wager, & Ochsner, 2012; Hazlett et al., 2005; Irle, Lange, & Sachsse, 2005; Lyoo, Han, & Cho, 1998). Later studies using whole-brain morphometric techniques such as voxel-based morphometry (VBM) and cortical thickness analysis have modified this picture to some extent. Thus, a 2019 meta-analysis of 13 studies (Yu et al., 2019 found a pattern of both grey matter volume/density decreases and increases in patients with BPD, the former being located in the bilateral ACC, other areas of the medial prefrontal cortex and the medial orbital frontal cortex, and the latter in the bilateral precuneus and the middle/posterior cingulate gyrus. A further cluster of volume reduction was noted in the right amygdala and hippocampal gyrus but not the hippocampus itself.

All the above studies were conducted on adult BPD patients, which makes them potentially susceptible to confounding by factors related to the evolution of the disorder over time. One such factor is the occurrence of major affective disorder in patients with the disorder, for which there is well-established evidence for an association (Fornaro et al., 2016; Grant et al., 2008; Zanarini, Frankenburg, Hennen, Reich, & Silk, 2004), and which itself is associated with brain structural changes. Other potential confounding factors include self-harming behavior, which has been found to be associated with brain structural change in BPD (Nenadić, Voss, Besteher, Langbein, & Gaser, 2020; Yi et al., 2024), and comorbid post-traumatic stress disorder, which has biological plausibility, particularly with respect to the hippocampus, although studies examining this factor in BPD have so far been equivocal on the question of brain structural changes (Rodrigues et al., 2011). One way of minimizing the influence of such confounding factors would be to examine adolescent patients with BPD. To date, however, there have been relatively few structural imaging studies in this age group, and they have had inconsistent findings. Thus, Chanen et al. (2008) examined regions of interest (ROIs) placed in the OFC, hippocampus and amygdala in 20 BPD teenagers and 20 matched controls and found a reversal of the normal (right > left) asymmetry in OFC volume. Examining the female patients in the same study (*N* = 15 and 15), Whittle et al. (2009) found reduced volume in the patients in an ROI placed in the ACC. Among voxel-based studies, Brunner et al. (2010) compared 20 adolescent patients with BPD and 20 healthy controls using VBM and found reduced grey matter in the dorsolateral prefrontal cortex (DLPFC) bilaterally and in the left OFC in the patients. Richter et al. (2014) found additional volume reductions in frontal regions, including the bilateral OFC and the right middle frontal cortex, as well as in bilateral superior parietal cortex, the hippocampus bilaterally, and the right amygdala in this sample using cortical thickness analysis. Finally, Yi et al. (2024) compared 52 patients with BPD aged 12–17 to 39 matched controls using whole-brain VBM. The patients showed decreased grey matter volume in the right calcarine cortex, the precentral cortex and the precuneus and in the left occipital, postcentral and supramarginal cortex, as well as in the right hippocampus and left putamen. Repeating the examination using cortical thickness analysis, Xiao et al. (2023) found reduced surface area in this sample in the left paracentral gyrus, left pars triangularis, right insula, and right lateral orbitofrontal gyrus.

In this study, we used VBM and automatic segmentation of hippocampal and amygdala volumes analysis to assess brain structural abnormalities in a relatively large sample of adolescent patients with BPD and matched healthy controls. We selected the patients to exclude comorbidities, and we also took advantage of the fact that a majority had not received drug treatment.

## Methods and materials

### Participants

The clinical sample consisted of 51 adolescents, 48 females and 3 males, diagnosed with BPD according to DSM-5 criteria, based on a structured interview with the Spanish version of the SCID-II (First et al., 1997). They were recruited from two outpatient resources for child and adolescent mental health in Barcelona, Vall d’Hebron University Hospital, and the Orienta Foundation. Exclusion criteria included left-handedness, age below 12 or above 18 years old, alcohol or substance abuse or dependence (excluding nicotine) in the last year, head injury with loss of consciousness, and standard exclusion criteria for MRI, such as the presence of metals within the body or pregnancy.

Another exclusion factor was a diagnosis of a comorbid psychiatric disorder. To screen for this, we used the Spanish version of Schedule for Affective Disorders and Schizophrenia for School Age Children-Present and Lifetime version (K-SADS) (Kaufman et al., 1997) in BPD patients and HS who were under 16 years of age, and the Spanish version of the Structured Clinical Interview for DSM-IV Axis I Disorders (SCID-I) (First, 1999) for participants aged 16–18. It has been argued that BPD can share symptomatic features with autistic spectrum disorder (ASD), particularly in the domain of social cognition (Allely, Woodhouse, & Mukherjee, 2023), and while these two diagnostic interviews do not address this disorder, we paid special attention to ensuring that participants included in the study did not have a history of neurodevelopmental alterations consistent with ASD.

Fourteen of the patients were taking psychotropic medication at the time of the study (13 antidepressants, in two combined with an antipsychotic, in three combined with a benzodiazepine and in three combined with an anticonvulsant; the remaining patient was taking a benzodiazepine). The remaining 37 patients were drug-naïve.

Forty-three healthy subjects (HS), 37 females, and 6 males, were recruited from the community, based on their similarity to the patients in age and IQ, as estimated using the Word Accentuation Test (Test de Acentuación de Palabras, TAP (Gomar et al., 2011). Exclusion criteria were the same as for the patients, and HS were also excluded if they reported having a first-degree relative with a psychiatric diagnosis.

All participants gave written informed consent prior to participation in accordance with the Declaration of Helsinki. Written informed consent was obtained from participants aged 18 years and from parents/legal guardians for all participants aged under 18. All the study procedures were approved by the Clinical Research Ethics Committee of Vall d’Hebron University Hospital [PR(AG)353/2015] and the Clinical Research Ethics Committee of Hermanas Hospitalarias del Sagrado Corazón de Jesús [PI15/02025]. The authors assert that all procedures contributing to this work comply with the ethical standards of the relevant national and institutional committees on human experimentation and with the Helsinki Declaration of 1975, as revised in 2008.

### Image acquisition and pre-processing

All subjects underwent a single MRI scanning session using a 3 T Philips Achieva scanner (Philips Medical Systems, Best, The Netherlands) at the Hospital de la Santa Creu i Sant Pau (Barcelona). High-resolution anatomical T1 volume was acquired using a TFE (Turbo Field Echo) sequence with the parameters: TR = 8.15 ms; TE = 3.73 ms; Flip angle = 8°; voxel size = 0.9375 × 0.9375 mm; slice thickness = 1 mm; slice number = 160; FOV = 240 mm.

#### Voxel-based morphometry

Structural data were analyzed with the FSL-VBM protocol (http://fsl.fmrib.ox.ac.uk/fsl/fslwiki/FSLVBM). An optimized VBM procedure (Good et al., 2001) was applied with FSL tools (Smith et al., 2004). First, structural images were brain-extracted and grey matter-segmented before being registered to the MNI 152 standard space using nonlinear registration (Andersson, Jenkinson, & Smith, 2007). The resulting images were averaged and flipped along the x-axis to create a left–right symmetric, study-specific grey matter template. Secondly, native grey matter images were nonlinearly registered to this study-specific template and ‘modulated’ to correct for local expansion/contraction due to the nonlinear component of the spatial transformation. The modulated grey matter images were then smoothed with an isotropic Gaussian kernel with a sigma of 4 mm. Finally, voxel-wise general linear model (GLM) was applied for group comparison of grey matter images between patients and controls. Statistical significance was assessed using permutation-based testing (5,000 permutations) implemented with the randomize function in FSL. Correction for multiple comparisons was performed using a threshold-free cluster enhancement (TFCE), a method that enhances cluster-like structures in the data without requiring an arbitrary cluster-forming threshold (Smith & Nichols, 2009). Age, sex, and intracranial volume (ICV) were included as covariates. Results were considered significant at a TFCE-corrected family-wise error (FWE) rate of *p* < 0.05.

#### Analysis of subcortical structures

To compare hippocampal and amygdala volumes between groups, we defined ROIs for these two structures using the Harvard–Oxford Subcortical atlas provided in the FSL package. Mean volumes for each subject in these ROIs were extracted from the individual GM-maps using FIRST (Patenaude, Smith, Kennedy, & Jenkinson, 2011) segmentation/registration tool part of FSL (Smith et al., 2004) that measures the volume of thalamus, caudate nucleus, putamen, pallidum, nucleus accumbens, hippocampus, and amygdala. Volumes were calculated for the left and right hippocampal and amygdala separately and compared between groups using age, TAP, sex, and ICV as covariates. Between-group comparisons were performed with the statistical JASP software (https://jasp-stats.org/). False discovery rate was used to correct for multiple comparisons (Benjamini & Hochberg, 1995).

Since the prevalence of BPD is higher in women (Skodol & Bender, 2003), a pattern that aligns with our sample of patients, we additionally conducted the above analyses on the subset of female participants.

## Results

### Demographic and clinical data

The 51 BPD patients and 43 HS were well-matched for age, sex, and TAP-estimated IQ, and they showed no significant differences in socioeconomic level or years of schooling (See Table 1).

**Table 1.** Demographic data for BPD patients and healthy subjects

	Healthy subjects (*n* = 43)	BPD patients (*n* = 51)	Statistic	*p*
Age (mean ± SD)	16.36 ± 0.93	15.93 ± 1.4	*t* = 1.67	0.10
TAP (mean ± SD)	19.12 ± 4	18.51 ± 3.8	*t* = 0.75	0.46
Sex (male/female)	6 M/37 F	3 M/48 F	*χ* ^2^ = 1.75	0.18
Socioeconomic level (median-range)	3 [1–5]	3 [1–6]	*Z* = 0.78	0.44
Years of schooling (mean ± SD)	10.69 ± 1.05	10.65 ± 1.56	*t* = 0.12	0.90

*Note:* Socioeconomic level was categorized as follows: 1 (High); 2 (Medium-High); 3 (Medium); 4 (Medium-Low); 5 (Low); 6 (Severe socioeconomics deficiencies).

The patients (mean 1630 ± 69 cm^3^) and controls (mean 1640 ± 55 cm^3^) showed no difference in ICV (*t* = 0.38; *p* = 0.70).

### Neuroimaging findings

#### VBM analysis

**Figure 1.**
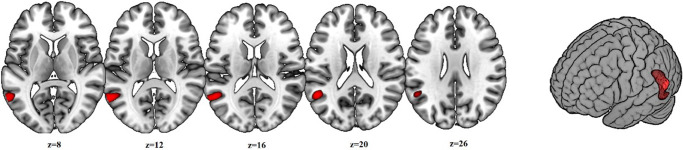
Cluster of reduced gray matter volume in the BPD patients in the voxel-based morphometry analysis.

At a TFCE-corrected *p*-value of 0.05, the BPD patients showed a cluster of reduced grey matter volume compared to HS in the left temporo-parietal junction (TPJ) (619 voxels; peak at MNI coordinates −58 −58 2, corrected-*p* = 0.009]) (see Figure 1).

#### Subcortical volumes

Amygdala and hippocampal volumes are shown in Table 2. There were no differences between the patients and controls in the volume of either structure.

**Table 2.** Volume of left and right amygdala and hippocampus for each group

	Healthy subjects(*n* = 43)	BPD patients(*n* = 51)	Statistic	*p* FDR-corrected (uncorrected)
Left amygdala (mean ± SD)	999 ± 131	1034 ± 172	*F* = 1.69	0.77 (0.20)
Right amygdala (mean ± SD)	1671 ± 166	1663 ± 284	*F* = 0.10	0.77 (0.75)
Left hippocampus (mean ± SD)	3650 ± 458	3531 ± 452	*F* = 0.61	0.77 (0.44)
Right hippocampus (mean ± SD)	3677 ± 535	3611 ± 457	*F* = 0.09	0.76 (0.77)

### Examination of the subsample of female patients and controls

The 48 BPD female patients remained well matched with the 37 healthy female controls for TAP-estimated IQ (mean HS =18.27 ± 3.48; mean BPD = 18.58 ± 3.81; *t* = 0.39, *p* = 0.69), but there was an age difference of 0.57 years, which was significant (mean HS = 16.43 ± 0.92; mean BPD = 15.86 ± 1.44; *t* = 2.11; *p* = 0.04). Both variables were therefore included as covariates in the analysis. Similarly, in the female subsample, there were no differences in ICV (mean of BPD patients = 1640 ± 55.5 cm^3^; mean of HS = 1640 ± 69.2 cm^3^; *t* = 0.05; *p* = 0.96), but as in the main comparison, this was also included as a covariate.

**Figure 2.**
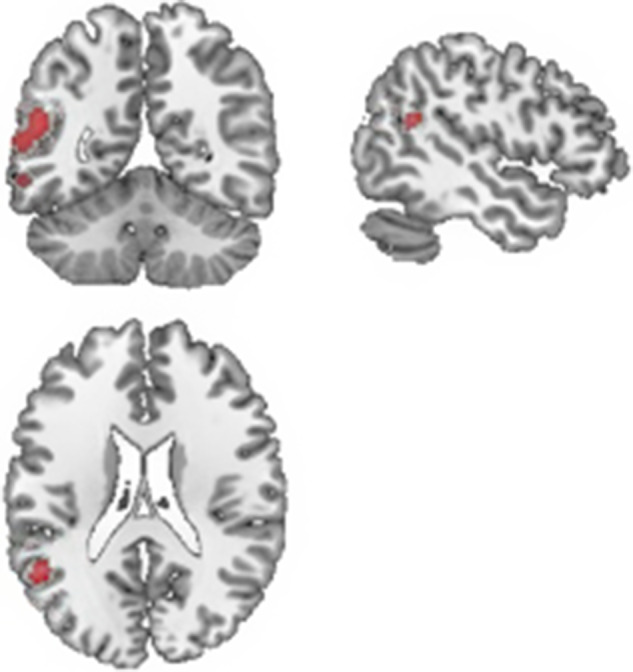
Cluster of major gray matter volume in female HS than in female BPD patients in the voxel-based morphometry analysis (p-TFCE corrected < 0.05).

*VBM analysis:* The findings were similar to those in the main analysis. There was a decrease in the volume of grey matter in the left TPJ (cluster of 131 voxels MNI: −60 −60 12, corrected-*p* = 0.03), although this was smaller than previously (see Figure 2).

*Subcortical volumes:* There continued to be no differences in the volume of the hippocampus or amygdala between the female BPD patients and the healthy controls (see Table 3).

**Table 3.** Volume of left and right amygdala and hippocampus in the female-only subsamples

	Healthy female subjects(*n* = 37)	BPD female patients(*n* = 48)	Statistic	*p* FDR-corrected(uncorrected)
Left amygdala (mean ± SD)	963 ± 120	1037 ± 167	*F* = 2.957	0.521 (0.356)
Right amygdala (mean ± SD)	1609 ± 132	1622 ± 175	*F* = 0.745	0.521 (0.391)
Left hippocampus (mean ± SD)	3565 ± 417	3492 ± 390	*F* = 1.06	0.521 (0.306)
Right hippocampus (mean ± SD)	3669 ± 502	3587 ± 460	*F* = 0.1598	0 .690 (0.690)

### Examination of the influence of drug treatment

The 37 drug-free patients remained well matched to the 43 HS on TAP-estimated IQ (mean HS = 19.1 ± 4.01; mean BPD = 18.4 ± 3.81; *t* = 0.87; *p* = 0.39), and in terms of sex distribution (HS = 6 male/37 female; BPD = 1 male/36 female; χ2 (Yates’ correction) = 1.90; *p* = 0.17, as well as in the ICV (mean HS = 1460 ± 18.50 cm^2^; mean BPD = 1380 ± 18.66 cm^2^; *t* = 1.12; *p* = 0.267). There was a trend level difference in age (mean HS = 16.4 ± 0.93; mean BPD = 15.8 ± 1.49; *t* = 1.97; *p* = 0.052). Age, sex, TAP-estimated IQ, and ICV were included as covariates.

**Figure 3.**
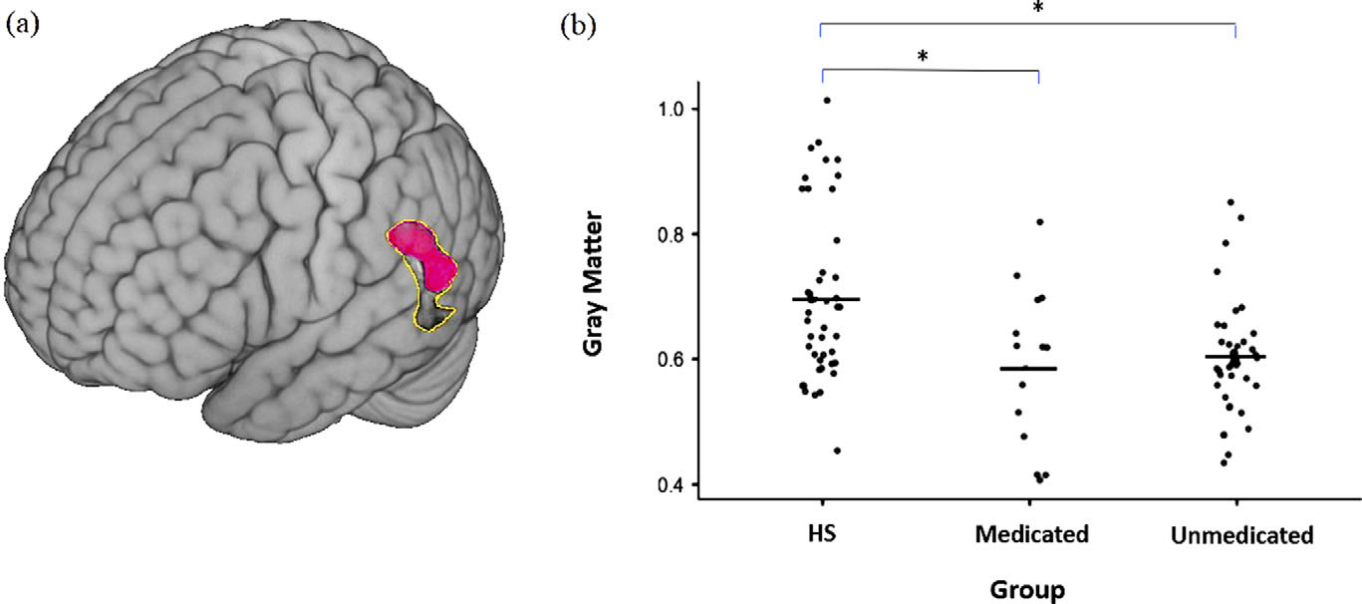
(a) Cluster of reduced gray matter in unmedicated patients compared to healthy subjects (magenta) overlaid on the reduction observed in the whole sample (main analysis) (yellow line); (b) Extraction of the ROI of patient-control difference obtained in the main analysis.

*VBM analysis:* Comparing the sample of unmedicated patients to the HS, a cluster of reduced grey matter volume continued to be seen in the left TPJ (270 voxels; MNI: −62 −60 12, corrected-*p* = 0.02, see Figure 3a).

A scatter plot of the individual values for the medicated and unmedicated patients and the HS within the ROI (619 voxels, 4952 mm^3^) did not suggest any difference in the distribution between the two patient groups (Figure 3b), and the comparison of the mean gray matter probability values found no significant difference between the treated and untreated BPD patients (mean treated = 0.60 ± 0.09; mean treated = 0.59 ± 0.13; *t* = 0.41; *p* = 0.68):

*Subcortical volumes:* There continued to be no differences in the volume of the hippocampus or amygdala between the unmedicated BPD patients and the healthy controls (see Supplementary Table S1).

## Discussion

In this study of 51 adolescent patients with BPD compared to 43 healthy controls, we found a single cluster of reduced grey matter volume in the patients in the left TPJ. This finding was present in the female-only subsample of patients and did not appear to be attributable to drug treatment, as volume reduction in this cortical region continued to be seen in never-treated patients.

Our finding of structural imaging change in the TPJ adolescent patients with BPD is novel and differs from those of existing studies, both in adults and adolescents. Thus, changes in the TPJ were not found in the 2019 meta-analysis of structural imaging studies in adult patients by Yu et al. (2019), even though they found a pattern of otherwise relatively widespread alterations of grey matter volume/density in patients with BPD. Nor did a relatively large study (*N* = 52 patients and 39 controls) in adolescents with BPD (Xiao et al., 2023; Yi et al., 2024) find changes in the TPJ or areas close to it, using either VBM or cortical thickness analysis. Accordingly, our findings need to be regarded as provisional and in need of replication. Nevertheless, they are of some potential theoretical interest, given that the TPJ has been argued to play a crucial role in understanding the beliefs of others (Samson, Apperly, Chiavarino, & Humphreys, 2004) and social cognition more broadly (Decety & Jackson, 2004; Eddy, 2016). Clearly, social interactions are at the heart of the disturbance in BPD, and so it would make intuitive sense that one of the first structural changes to appear in the disorder would be in an area that is implicated in such processes.

If our finding of TPJ volume reduction in BPD turns out to be replicable, the question still needs to be asked as to how this could be present in adolescent but not adult patients, according to current evidence (eg the meta-analysis of Yu et al. (2019)). One possibility here, as noted in the Introduction, is that a simple picture of brain structural change in adult patients might become progressively complicated by the cumulative effects of comorbid major affective disorder, traumatic events and/or episodes of self-harm on the brain. However, this would not in itself explain why volume reduction in the TPJ would no longer be evident in adult patients. For this, it would be necessary to evoke additional processes of brain maturation. This is in some ways an attractive possibility, since some theories propose that BPD ultimately has a developmental origin and/or implicates changes occurring during the transition from adolescence to adulthood (e.g., Newton-Howes, Clark, & Chanen, 2015; Videler, Hutsebaut, Schulkens, Sobczak, & Van Alphen, 2019). Some evidence provides a degree of general support for such a view: Kimmel et al. (2016) meta-analyzed nine VBM studies that examined grey matter volume abnormalities in BPD and found that age showed a significant correlation with an increase in grey matter volume in the superior parieto-occipital gyrus, with younger patients starting at a lower volume compared to controls. As age increased, there was also a decrease in volume in the right amygdala. However, this meta-analysis did not identify progressive changes in the TPJ.

Our failure to find evidence of hippocampal or amygdala volume reductions in adolescent patients with BPD goes against two often-cited meta-analyses of ROI studies in adult patients (Nunes et al., 2009; Ruocco et al., 2012). However, it is fair to say that these findings may not be as robust as originally thought. Thus, as noted in the Introduction, Yu et al. (2019) found a cluster of volume reduction involving the right amygdala and hippocampal gyrus in their meta-analysis of whole-brain voxel-based studies but not the hippocampus itself. It may also be relevant in this respect that our group failed to find changes in amygdala or hippocampal volume in a large sample of adult patients with BPD (*N* = 76 patients and 76 healthy controls) (Aguilar-Ortiz et al., 2018), based on both VBM and ROI analysis. Findings are currently divided in studies of adolescent patients: one study found no volume changes in amygdala and hippocampus volume in 20 adolescent with BPD compared to 20 healthy controls using a manual tracing approach (Chanen et al., 2008), whereas another reported decreased volume in bilateral hippocampus (HPC) and the right amygdala in 20 female BPD patients compared to 20 matched controls, using automated measurement (Richter et al., 2014).

In conclusion, this study finds evidence of decreased grey matter volume in the left TPJ, a region that is believed to be important for social cognition, in adolescent patients with BPD. As the volume reduction was present before adult life and present in patients without comorbidities, as well as in the subsample who were drug-free, this finding might be relevant to understanding the developmental trajectory of the disorder. Some limitations should be noted. Our sample was predominantly female, and so the finding may not apply to BPD as it is seen in males. Also with reference to applicability, our findings were in patients without axis I comorbidity, which may affect their generalizability to BPD as encountered in everyday clinical settings. Additionally, although we matched participants for age and sex, we did not assess pubertal stage, which could influence brain maturation independently of chronological age, and so is a factor of potential relevance in an adolescent sample. While we excluded patients with substance use disorder, we did not collect data on recreational drug/alcohol use. Finally, the study was cross-sectional in nature, and a follow-up study would be necessary to confirm the suggestion that changes in brain morphology are dynamic in the disorder.

## Supporting information

Salgado-Pineda et al. supplementary material Salgado-Pineda et al. supplementary material

## Supplementary material

The supplementary material for this article can be found at http://doi.org/10.1017/S0033291725101645.
