# Supplementary material for: Early structural brain abnormalities in borderline personality disorder
Source: Psychol Med. 2025 Oct 13;55:e311. doi: 10.1017/S0033291725101645 (PMC12551577; doi:10.1017/S0033291725101645)
Supplement: Salgado-Pineda et al. supplementary material [file S0033291725101645sup001.docx]

**Supplementary Data**

*Table S1: Volume of left and right amygdala and hippocampus for the subsample of unmedicated patients and the sample of healthy subjects*

|  | **Healthy Subjects**  **(n=43)** | **Unmedicated BPD Patients**  **(n=37)** | **Statistic** | **p FDR-corrected**  **(uncorrected)** |
| --- | --- | --- | --- | --- |
| **Left Amygdala**  (mean ± SD) | 999 ± 131 | 1026 ± 145 | F= 2.449 | 0.488  (0.122) |
| **Right Amygdala**  (mean ± SD) | 1671 ± 166 | 1649 ± 164 | F= 0.023 | 0.88  (0.880) |
| **Left Hippocampus** (mean ± SD) | 3650 ± 458 | 3532 ± 400 | F= 0.147 | 0.88  (0.702) |
| **Right Hippocampus** (mean ± SD) | 3677 ± 535 | 3642 ± 471 | F= 0.036 | 0.88  (0.850) |
